# Discriminate Use of Gold

**Published:** 1887-04

**Authors:** S. B. Palmer

**Affiliations:** Syracuse, N. Y.


### DISCRIMINATE USE OF GOLD.

BY S. B. PALMER, M. D. S., SYRACUSE, N. Y.

READ BEFORE A UNION MEETING OF THE 5TH, 6TH, 7TH AND 8TH DISTRICT DENTAL SOCIETIES OF NEW YORK, HELD IN ROCHESTER, OCTOBER 26 AND 27, 1886.

Having consented to present a paper on this occasion, I could think of nothing more needed in the profession than a few simple suggestions relative to the discriminate adaptation of gold to the various conditions of dentine, in filling teeth. Time and experience may furnish such knowledge; but when the young operator seeks definite instruction upon this point, either in text books or dental literature, he will find few practical suggestions and rarely any scientific teachings. Formerly, gold was considered the only suitable filling material, and arguments to the contrary are of recent origin. Professional advancement, at present, calls for operations upon a class of teeth which formerly would have been doomed to the forcep. This does not conflict with the fact that gold is still the best material to use for the great majority of fillings. There are cases and conditions, however, where it should not be employed. When, where, and how it is advisable, are questions for our consideration. One short paper cannot give in detail all the combinations and circumstances which are presented in practice. Fortunately, they differ in degree rather than in principle, and once the laws are known, good judgment will guide operations. Therefore, we draw illustrations from extremes, and give to you the outcome of all our incoming knowledge from any and every source.

Filling teeth involves two principles; both must be understood to insure success. First, the operator needs to be a master mechanic, so far as the preparation of cavities and the manipulation of gold is concerned. Second, he should understand the relations of gold to the varied conditions of tooth-structure, in order to anticipate probable results. Each operation may be regarded a cause, which is related by law to positive effects.

The day of writing this I examined the teeth of a patient who had a dozen or more fillings, all gold, and in good order. My attention was called to one in a lateral incisor which I had inserted thirty years ago. The patient's teeth have been under my care ever since, a few fillings only having been replaced. From first to last there was no demand for plastic fillings, and no excuse for failures. With this case before us, let us understand the principles of success. All understand that the teeth referred to were of excellent quality; but we want to know something of the difference between dense teeth and soft teeth, also what chemical action may be expected from the introduction of gold plugs in soft teeth. Good normal teeth usually contain about one-third of organic or animal matter, and two-thirds of mineral or inorganic matter. It so happens that about this proportion of inorganic, non-conducting material, reduces vitality to a degree below severe inflammatory action, at least below the danger of devitalization from such action; consequently, instead of destruction of the surface in contact with the filling, the excitement caused by thermal changes stimulates the bone-producing agents, and increased calcification is the result, by the same process witnessed in the grinding down of the oral teeth by attrition. We often find the walls hardened beneath gold fillings, but we look in vain for this process in poorly calcified teeth, unless some less-conducting material lines the cavity.

Thus far we have considered the better class of teeth. This class, when filled, come under a positive law for preservation. Now, please consider the other extreme—say the teeth of a child from ten to fourteen years of age, with numerous lateral cavities in the incisors. I need not say that such teeth are soft and far below the average. The presence of decay indicates the true condition. They resemble cartilage nearly as much as bone. Does any one pretend that gold is a suitable material for filling this class of teeth ? The fact that occasionally we see it so used leads us in charity to believe so. We have in this class an utter change of conditions, which, if treated like those already noted, would give opposite results, and which in time would be recorded as failures. And why ? In the fully matured tooth the organic matter is given as twenty-eight parts in one hundred, against seventy-two parts of lime, etc. Thus the pulp and spaces occupied by the vital fluids are so contracted and protected that unless cavities reach very near the pulp, thermal changes cause only temporary inconvenience.

With the poorly calcified organ, the large pulp and increased vitality renders the preparation of a cavity painful, and the packing of gold exceedingly difficult. This is not all. Such operations are followed by thermal disturbances. Vitality will endure a given amount of irritation, excitement or inflammation, and yet set to work to ward off the enemy and repair damages. Beyond a certain strain the work is abandoned. Devitalization first occurs in the dentine, merely upon the surface in contact with the filling. It is first manifest to the eye by the blue or gray shade reflected through the enamel. When disintegration sets in, the decomposed dentine becomes a conductor and positive element, the gold being negative.

With this understanding of the two extreme conditions, it will readily be understood why former discussions upon the "Electro-chemical Theory" seemed so conflicting and unsatisfactory. The opposition presented the one condition most favorable to overthrow of the theory, which was substantially this : "Tooth-bone, being a non-conductor, is not an electrolite, therefore is not decomposed by galvanic currents." This being true, it was received by the general reader as the *whole* truth. On the other extreme, abnormal tooth-bone, that in which the organic matter predominates, *is* a conductor, and like the portions of animal matter deposited from food, as well as the oral fluids, is an electrolite, and is decomposed, the gold in contact being the negative element.

One great hindrance to dental progress is the lack of discrimination. Chemical results are in accordance with fixed laws. If we desire certain results, we must certainly understand the laws and conditions by which such results are possible.

My present practice is based upon the following conclusions: That gold may be used with safety in all teeth of normal structure and density; also in devitalized teeth of like structure, even though the color might indicate a lower grade. For teeth answering the above conditions, all things considered, gold is superior to all other filling materials.

Gold should not be packed upon highly sensitive dentine, as thermal changes occur which may be avoided by filling with gutta-percha, even for a few days or weeks.

Gold should not be malleted into teeth while sensitive from recent wedging. This condition presents a three-fold danger; the dentine pulp and periosteum are unduly inflamed. A retaining wedge of gutta-percha, worn from six to eight days, removes all trouble.

Gold should not be inserted or packed upon dentine so poorly calcified that the excavator fails to give the sound and sense of cutting bone, because the surface in contact with the plug will lose vitality, decompose, and eventually turn dark.

Gold should not be malleted upon dentine below normal structure. Bruised dentine will decompose, and not harden, by calcification under a filling.

The utility of gold may be greatly extended by the lining of cavities with a varnish, and by combination with various plastic materials, through methods generally known and practiced. Its combination with tin is of sufficient importance to receive special consideration.

There are two distinct methods involved in the combination. Each will give positive results when specific conditions are observed, any change of conditions leading to seeming contradictions.

The substance of the first condition or method has been published and commented upon. It applies to filling large cavities, generally in molars, as a substitute for amalgam, the object being to form an alloy of gold and tin by chemical action upon the surface of the plug. Such fillings resemble amalgam in color and hardness, without the disadvantage of either shrinkage or discoloration of the dentine. To obtain an alloy it is essential that the two metals be evenly distributed throughout the plug. There may be an excess of gold at any point without injury, but the tin foil must not exceed one or two thicknesses of No. 3 foil, for the reason that the molecular action desired is limited or confined near the surface of the two metals in contact. An excess of tin greater than can be fused with the gold is chemically dissolved, leaving a corresponding furrow or pit in the plug.

It should be borne in mind that this union or combination depends upon moisture. That is, a filling well packed in a good cavity remains the same except upon the surface. If it be upon the grinding portion, where the filling is self-cleansing, there seems to be little difference from a first-class tin filling, but on approximate, buccal or lingual surfaces, especially where little care is bestowed, the surface of the plug becomes hard and indestructible, which renders this preparation of great value for submarine operations. I prepare the material for this filling by placing a sheet of No. 3 tin upon a No. 4 sheet of gold. This will usually fill a cavity. If thought to be sufficient, cut through the leaves and place the gold of one-half upon the tin of the other, thus giving four thicknesses, from which blocks or ribbons can be cut, the latter being rolled into cylinders. This preparation makes an excellent foundation for large gold fillings at the cervical borders of approximate cavities, where the matrix can be used, as there is no danger that the tin will be dissolved by chemical action. Cutting the foil unites the edges and secures the position of the layers while packing.

The second method is applicable for any filling in the posterior teeth, and much better than amalgam against frail, transparent walls of bicuspids. It possesses all the advantages of a gold filling in appearance through the enamel, as well as the benefits of tin in packing and closely adapting the gold to the dentine and enamel. Many gold fillings fail because the contact is not absolute when the filling is completed. Some patients are prejudiced against amalgam, who would like to compromise, if possible, between the expense of amalgam and gold. This can be accomplished by the use of tin and gold. The greatest benefit to the operator consists in the saving of time.

Unlike the conditions already mentioned, this method applies to teeth of normal structure, where there is no occasion for the antiseptic properties which are afforded by tin and amalgam, consequently the gold is intended to cover the tin, and of course the cavity would be lined with gold. The preparation of material is as follows: Cut number four gold into strips from three-fourths to one inch wide; also prepare number three tin foil in the same manner. Roll the tin into a rope and untwist it, and this will leave a pliable roll of tin. Anneal the gold, taking care not to melt it. Place the gold upon a napkin and the roll of tin upon it, bring the edges of the gold up over the tin and lap them, and the tin will be completely covered. This may be used as cohesive gold. Pellets may be made by cutting the gold into squares of about one inch, and making a loose pellet of tin ; anneal the gold and enclose the tin in it. With either rope or pellet, fill as much or little of the cavity as desired. Cohesive gold may be added to finish, and when properly done the appearance is that of a first-class cohesive gold filling, and much better than the latter, as the gold is more perfectly pressed against the walls. One thing should not be overlooked when the tin has been used. If cohesive gold is to be added, let the first piece be of one or two thicknesses of annealed gold, packed with an instrument of four well-defined points, by which the gold will receive a firm mechanical attachment to the tin foundation. If this be not done the gold covering is liable to be drawn from the tin, leaving it bare and non-cohesive.

It is well to remove the gold from the pieces left after filling, because fresh annealing is necessary, and also because confusion might arise from allowing such foil to get mixed with that in common use. It is a pleasure to lay foundations of this combination for large fillings where there is danger of the intrusion of moisture, or when it is difficult to anchor the pellets in place. By judicious use of gold and tin, gold may be greatly helped to preserve teeth.

The foregoing suggestions have been taken from daily practice. Whether they are worth serious attention is left for your judgment. Bear in mind that the result of every operation is determined by chemical laws. Change of circumstances gives different results. Neither mechanical skill nor good intentions can atone for ignorance of natural laws. Therefore, I say to the young men, strive to adopt a scientific and systematic method of practice. Note conditions and record results, for this will aid in accurate discrimination. Correct diagnosis is prerequisite to a judicious selection and adaptation of filling materials to the varied conditions of teeth.

